# Supplementary material for: Raman and Photoemission Spectroscopic Analyses of Explanted Biolox® Delta Femoral Heads Showing Metal Transfer
Source: Materials (Basel). 2017 Jul 3;10(7):744. doi: 10.3390/ma10070744 (PMC5551787; doi:10.3390/ma10070744)
Supplement: Supplementary file 1 [file materials-10-00744-s001.pdf]

materials MDPI

Supplementary Material

Raman and photoemission spectroscopic analyses of explanted Biolox® delta femoral heads showing metal transfer

Paola Taddei [1*], Eleonora Pavoni [1] and Saverio Affatato [2]

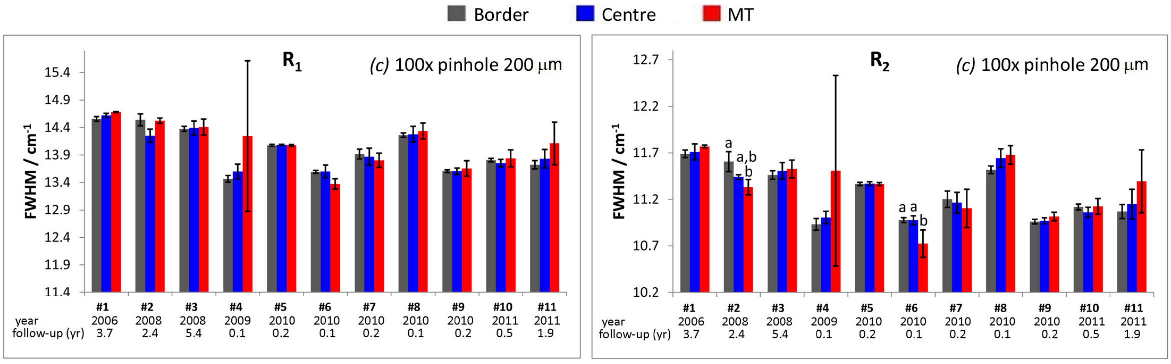

Figure S1. Average FWHM values (FWHM ± standard deviation) of R_1 and R_2 bands obtained from the fitting of the emission spectra recorded under optical conditions *(c)* in different areas of the explanted Biolox® delta femoral heads. When present, different letters on the histogram bars indicate significant differences among the areas of each retrieval.

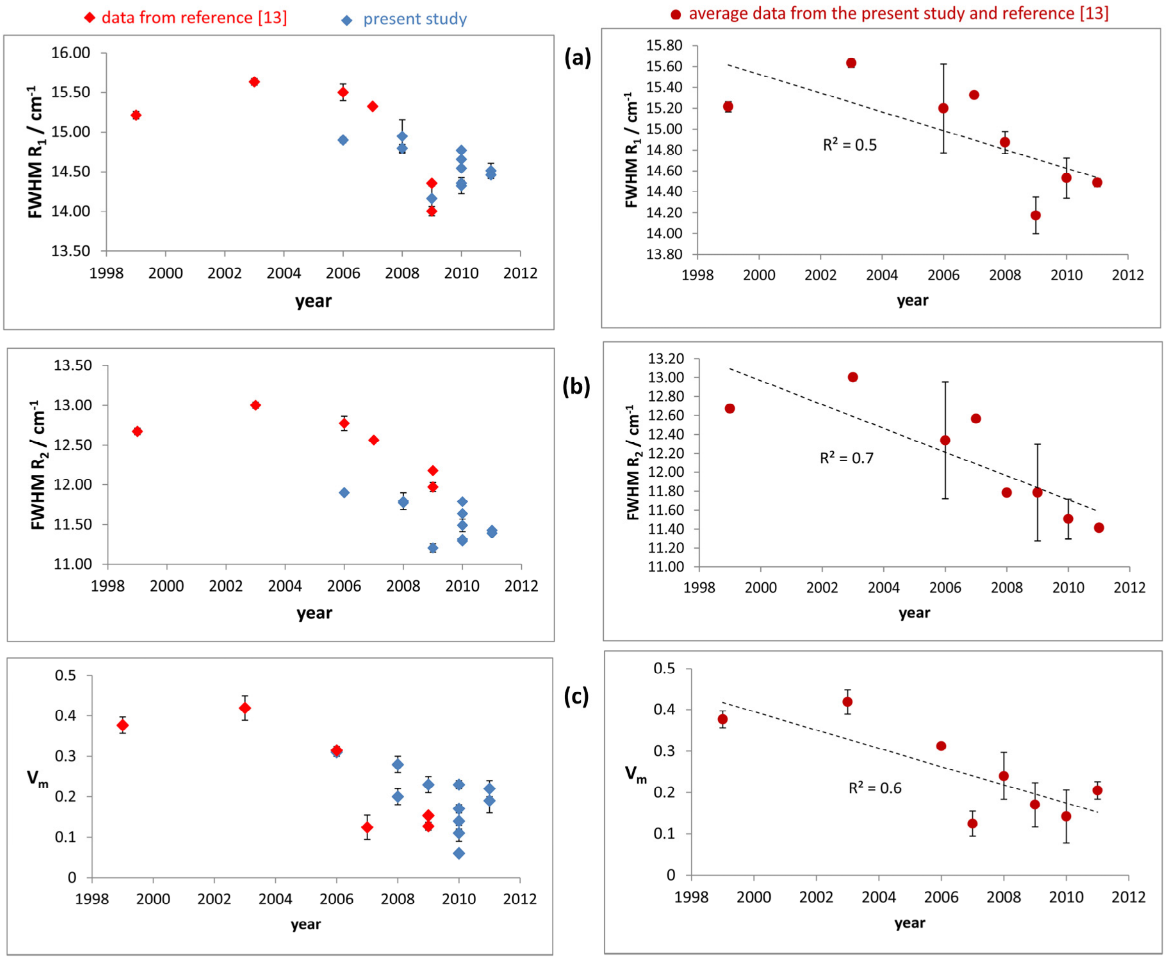

Figure S2. Trend of the FWHM of R_1 (**a**) and R_2 (**b**) photoemission bands and V_m monoclinic zirconia content (**c**) measured in the border control areas of the retrievals as a function of the implantation year. Left: single retrievals data from the present study and reference [13]; right: average data from the present study and reference [13].

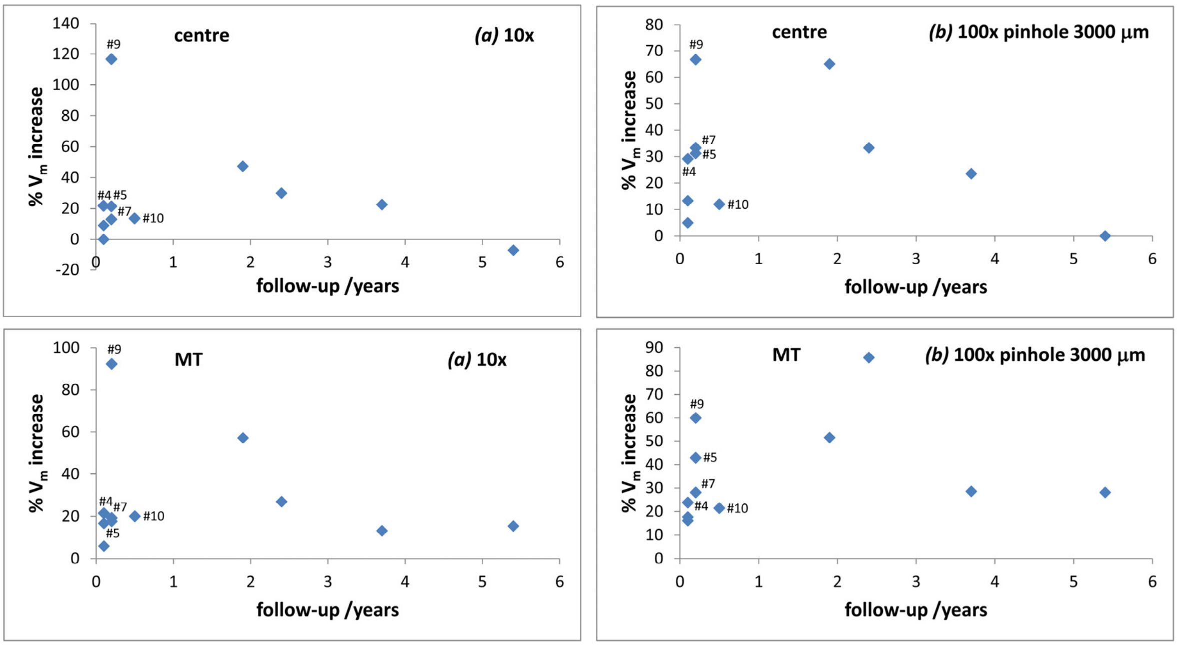

Figure S3. Trend of the % V_m increase observed in the centre and MT areas of the retrievals under study as a function of follow-up. The % V_m increase was calculated with respect to the control border of the same femoral head, under the same optical conditions ((*a*) or (*b*)).

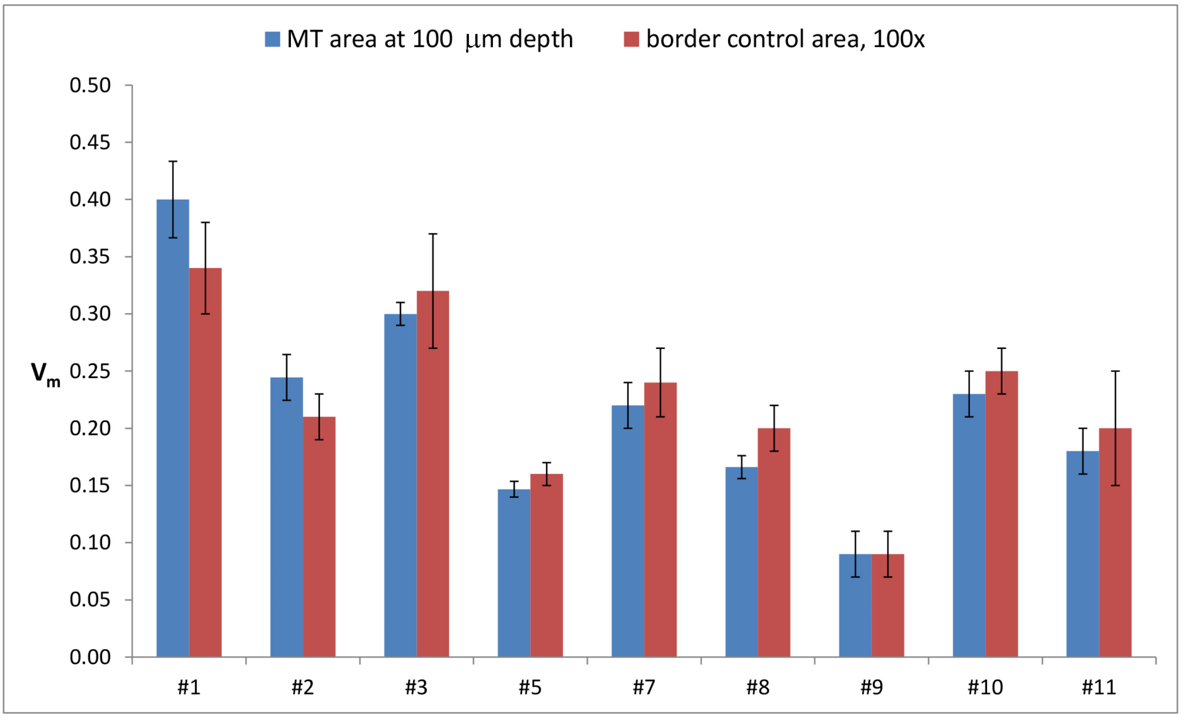

Figure S4. V_m values measured 100 μm below the surface of retrievals #1, #2, #3, #5, #7, #8, #9, #10, #11 in depth profiling analyses and in their border control area under optical conditions (*b*).
